# Dentists United to Extinguish Tobacco (DUET): a study protocol for a cluster randomized, controlled trial for enhancing implementation of clinical practice guidelines for treating tobacco dependence in dental care settings

**DOI:** 10.1186/1748-5908-9-25

**Published:** 2014-02-21

**Authors:** Jamie S Ostroff, Yuelin Li, Donna R Shelley

**Affiliations:** 1 Department of Psychiatry and Behavioral Sciences, Memorial Sloan-Kettering Cancer Center, 1275 York Avenue, New York, NY 10022, USA; 2 Department of Population Health, New York University School of Medicine, 227 East 30th Street, 7th floor, New York, NY 10016, USA

**Keywords:** Dental care, Clinical practice guidelines, Treatment of tobacco dependence, Tobacco cessation

## Abstract

**Background:**

Although dental care settings provide an exceptional opportunity to reach smokers and provide brief cessation advice and treatment to reduce oral and other tobacco-related health conditions, dental care providers demonstrate limited adherence to evidence-based guidelines for treatment of tobacco use and dependence.

**Methods/Design:**

Guided by a multi-level, conceptual framework that emphasizes changes in provider beliefs and organizational characteristics as drivers of improvement in tobacco treatment delivery, the current protocol will use a cluster, randomized design and multiple data sources (patient exit interviews, provider surveys, site observations, chart audits, and semi-structured provider interviews) to study the process of implementing clinical practice guidelines for treating tobacco dependence in 18 public dental care clinics in New York City. The specific aims of this comparative-effectiveness research trial are to: compare the effectiveness of three promising strategies for implementation of tobacco use treatment guidelines—staff training and current best practices (CBP), CBP + provider performance feedback (PF), and CBP + PF + provider reimbursement for delivery of tobacco cessation treatment (pay-for-performance, or P4P); examine potential theory-driven mechanisms hypothesized to explain the comparative effectiveness of three strategies for implementation; and identify baseline organizational factors that influence the implementation of evidence-based tobacco use treatment practices in dental clinics. The primary outcome is change in providers’ tobacco treatment practices and the secondary outcomes are cost per quit, use of tobacco cessation treatments, quit attempts, and smoking abstinence.

**Discussion:**

We hypothesize that the value of these promising implementation strategies is additive and that incorporating all three strategies (CBP, PF, and P4P) will be superior to CBP alone and CBP + PF in improving delivery of cessation assistance to smokers. The findings will improve knowledge pertinent to the implementation, dissemination, and sustained utilization of evidence-based tobacco use treatment in dental practices.

**Trial registration:**

NCT01615237.

## Background

### Evidence-based approaches for the treatment of tobacco dependence exist

The 2008 United States (US) Public Health Service (PHS) Guideline, *Treating Tobacco Use and Dependence*, provides strong evidence that provider delivery of tobacco dependence treatment, including cessation pharmacotherapy and brief counseling, can produce significant and sustained reductions in tobacco use and should be delivered to all smokers seeking routine healthcare [1]. Provider adherence to the PHS Guideline recommendations involves asking all patients about tobacco use, advising smokers to quit, assessing readiness to quit, providing cessation assistance, and arranging follow-up (the so-called 5As) [1].

### Inadequate adoption of evidence-based treatment contributes to disparities in tobacco related illnesses

Despite the existence of effective tobacco dependence treatments, inadequate adoption, particularly among low-income and ethnic/racial minority smokers, has contributed to growing disparities in smoking prevalence and tobacco-related illness [2-4]. Citing persistent missed opportunities to promote tobacco cessation, the Institute of Medicine’s (IOM) report [5], ‘Ending the Tobacco Problem: A Blueprint for the Nation,’ called for greater efforts to implement effective tobacco cessation interventions in healthcare settings. These recent health policy reports highlight the need and potential public health value of reducing health disparities through dissemination of evidence-based tobacco cessation interventions in healthcare delivery systems serving low-income and other high-risk smokers [5,6].

### Dental healthcare settings are an important but underutilized venue for tobacco use treatment

Dental care settings have several advantageous features for delivery of tobacco cessation treatment including: broad reach with 62.8% of 18 to 64 years olds reporting at least one annual dental visit [7]; access to patients who do not receive other healthcare services (10% of dental patients do not regularly see a physician) [8]; the dental team routinely provides preventive services; and controlled trials have demonstrated the efficacy of dental office-based cessation interventions [9]. Moreover, dental professionals have a credible role in providing tobacco cessation advice and treatment because of the oral hazards of tobacco use. A recent national survey found that 89% of dentists and 96% of dental hygienists reported that treating tobacco use was an important professional responsibility [10]. There are approximately 475 federally-funded, community or neighborhood health centers with dental clinics and another 250 community dental clinics throughout the US [11]. These community dental health centers serve predominantly low-income populations known to have a high prevalence of smoking [12]. The potential impact of implementing tobacco clinical practice guidelines in these public health dental clinics is substantial [9].

### Treatment of tobacco dependence in dental care settings remains suboptimal

Although national surveys indicate that dental providers are increasingly screening for tobacco use and offering brief advice, full adherence to the PHS guidelines is inconsistent with only 10% to 25% dental care providers routinely delivering cessation assistance (*e.g.*, cessation pharmacotherapy prescriptions and/or referral for cessation counseling) [10,13]. These findings are consistent with international findings demonstrating that treatment of tobacco dependence in dental settings is lacking [14]. Dentists most often cite lack of training and inadequate reimbursement to explain their limited provision of tobacco cessation interventions [15]. PHS guideline implementation is likely affected by both provider attitudes and organizational priorities that impact provider behavior [1,13,16-18].

Closing the gap between research and practice is stymied by the paucity of research on systems changes needed to implement tobacco treatment in routine dental care. Drawing from a burgeoning implementation science literature, including studies focusing on implementing tobacco cessation counseling in dental care settings [19,20], the current research protocol will compare the cumulative benefit of three, promising systems-level strategies: staff training, practice facilitation and clinical reminders (CBP), provider feedback and pay-for-performance (financial incentives), that have been widely endorsed by an IOM report and the PHS Guidelines [1,21,22].

### Staff training and clinical reminder systems (CBP)

The PHS Guideline strongly recommends staff training, clinical reminder systems, referral pathways and other practice supports as CBP for screening and treating tobacco dependence in all healthcare settings [1,16].

### Performance feedback (PF)

In recent randomized trials conducted in primary medical care settings, clinical audit and feedback with regard to tobacco treatment performance have been associated with a twofold increase in providers’ adherence to cessation assistance and referral to cessation quitlines [16-18,21,23-25]. The potential value of these strategies has not yet been fully examined in dental practice. A single arm, pilot study tested the feasibility and promise of implementing CBP plus provider feedback in six public dental clinics in New York City [26]. The main outcome measure—provider adherence to tobacco use treatment guidelines—was assessed by auditing a random selection of dental chart documentation pre and post-adoption of this implementation strategy. Following implementation of CBP and PF, providers were significantly more likely to offer advice (28% pre, 49% post), assess readiness to quit (18% pre, 30% post), and offer assistance (7% pre, 16% post) [26,27].

### Pay for performance (P4P)

P4P or providing financial incentives for meeting predetermined performance goals has attracted much interest as a strategy to improve quality of care [28,29]. The European Workshop on Tobacco Use Prevention and Cessation for Oral Health Professionals emphasized the importance of appropriate compensation (incentives) of tobacco use treatment [30]. Several studies have demonstrated a positive association between P4P and adherence to recommended tobacco use treatment [31-33], including increased referrals to statewide tobacco quitline services [31].

Using a three-arm cluster randomized controlled trial design, the overall objective of this protocol is to compare the effectiveness of three promising implementation strategies designed to address organizational and provider level challenges for effective treatment of tobacco dependence. Guided by a multi-level, conceptual framework that emphasizes both provider beliefs and organizational characteristics, the trial will be conducted in 18 public dental care clinics in New York City and information about implementation outcomes and processes will be captured with multiple data sources (patient exit interviews, provider surveys, site observations, chart audits, and semi-structured provider interviews) in order to examine factors that influence the implementation process.

The specific aims of this comparative effectiveness research trial are to:

1. Compare the effectiveness and cost of three promising strategies for implementation of the PHS tobacco use treatment guidelines on dental provider delivery of cessation assistance:

a) Staff training and CBP;

b) CBP + provider PF; and

c) CBP + PF + provider reimbursement for delivery of tobacco cessation treatment (pay-for- performance; P4P)

2. Use a mixed-methods approach to examine potential theory-driven mechanisms at the organizational and provider level hypothesized to explain the comparative effectiveness of three strategies for implementation;

3. Identify baseline organizational factors that influence the implementation of evidence-based tobacco use treatment practices in dental clinics.

The central hypothesis is that the value of these promising implementation strategies is additive and that instituting all three strategies (CBP, PF, and P4P) will be superior to CBP alone and CBP + PF in increasing delivery of cessation assistance to smokers. The ultimate goal is to provide critical new knowledge to facilitate the widespread implementation, dissemination and sustained utilization of evidence-based tobacco use treatment strategies in dental practices.

## Methods

### Overview of study design

**Figure 1:**
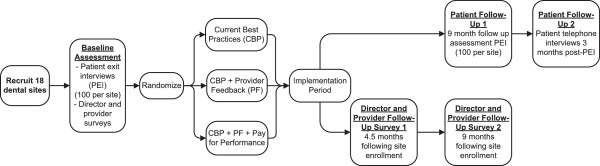
Study design.

As shown in Figure 1, a three-arm cluster randomized controlled trial will examine the tobacco treatment guideline implementation process and compare the cost and effectiveness of three implementation strategies: Staff training and CBP in implementing PHS Guidelines; CBP + PF; and CBP + PF + pay-for-performance (provider reimbursement for tobacco cessation treatment delivery). Guided by Organizational Change Theory and the Theory of Planned Behavior (TPB) [34-37], multi-level factors that facilitate or impede the implementation process will be identified. The primary outcome is improvement in dental provider delivery of tobacco cessation treatment. Secondary outcomes are cost per quit, use of tobacco cessation treatments, quit attempts and smoking abstinence. A mixed methods (survey, semi-structured interview, chart audit, site observation and NYS Quitline referral report) approach will be used to examine implementation processes (aim two).

### Conceptual framework

**Figure 2:**
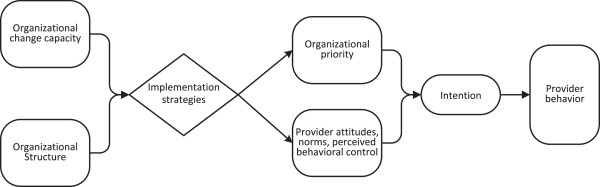
Conceptual framework.

This study’s multi-level, conceptual framework and corresponding selection of process measures are informed by both the Organizational Change Model [37] for studying organizational priority and practice improvements and the Theory of Planned Behavior for understanding the role of provider attitudes, norms, perceived behavioral control, and intentions on provider behavior [34]. Guided by these two complementary theoretical models and prior reviews of the implementation science literature [35,36,38-42], the proposed implementation strategies are hypothesized to operate at both the individual health provider level and the organizational level (see Figure 2).

Solberg posits that the primary organizational factors predicting implementation are organizational change capacity, organizational priority (*i.e.*, extent to which the organization supports implementation of tobacco use treatment guidelines relative to other priorities) and effective system changes [25,37]. Implementation strategies (*i.e.*, system changes) are also hypothesized to influence dental provider attitudes, subjective norms, and perceived behavioral control, all constructs from the TPB [34,43-45]. TPB is a robust theoretical model that has been applied successfully to explain clinical behavior change among healthcare providers [46-50].

As shown in Table 1, arm one (*e.g.*, provider training, chart reminders, practice support for referral to the Quitline or other local cessation programs) is hypothesized to impact perceived behavioral control. The addition of provider performance feedback, (arm two) is hypothesized to enhance provider adherence to the tobacco use treatment guidelines through changes in subjective norms and attitudes. Arm three, which adds financial incentives, is expected to impact the relative priority that organizations and providers attribute to tobacco use treatment by addressing a frequently cited organizational barrier (*i.e.*, lack of reimbursement).

**Table 1:** Theory-driven mechanisms hypothesized to explain the effect of the implementation strategies

**Implementation strategy**	**Theory-driven hypothesized mechanism(s) of change**
Arm one Staff training and current best practices (CBP)	Increases perceived behavioral control
Arm two CBP + audit and performance feedback (CBP + PF)	Increases perceived behavioral control AND modifies providers’ subjective norms and attitudes
Arm three CBP + PF + pay for performance (CBP + PF + P4P)	Increases perceived behavioral control, provider attitudes AND increases organizational change priority

### Description of intervention conditions

Table 2 summarizes the implementation strategy components for each treatment arm.

**Table 2:** Specific implementation strategies by treatment arm

**Implementation strategy components**	**Arm one CBP**	**Arm two CBP + Performance Feedback (PF)**	**Arm three CBP + PF + Pay for Performance (P4P)**
1. Staff training on PHS Guideline	X	X	X
2. Chart reminder	X	X	X
3. Cessation treatment workflow	X	X	X
4. Quitline referral system	X	X	X
5. Tool Kit	X	X	X
- Letter from the Health Commissioner			
- Recommended clinical pathway			
- Cessation medication guide			
- Provider guide for delivering cessation counseling			
- Patient education booklets on the oral effects of tobacco use			
- Quitline wallet cards			
- Report on oral health in NYC			
- Waiting room poster			
6. Smokers’ chart audit and quarterly Performance Feedback reports		X	X
7. Pay for Performance			X

### Arm one: staff training and CBP

All dental clinic sites will receive CBP for training and onsite technical assistance in promoting adoption of clinical practice guidelines for treating tobacco dependence. In New York state (NYS), the scope of dental practice includes tobacco cessation treatment. The CBP tobacco use treatment protocol that will be implemented is consistent with the PHS recommended guidelines. The dental care team will assess smoking status, deliver advice to quit, assess readiness to quit, provide patient education materials, provide a prescription for cessation pharmacotherapy and/or make referral to the NYS Quitline, and document findings and treatment plan on the chart system. In NYS, all cessation medications except the nicotine lozenge, are covered by Medicaid and the Quitline provides eligible clients at least a two-week supply of free nicotine patches. Details of CBP are summarized as the following.

#### Staff training

All clinical (*i.e.*, dentists, dental hygienists, and/or dental assistants) and support staff will be trained in the use of the intervention protocol during a one-hour, slide presentation. The didactic training is based on the PHS guideline [1] and will include content on prescribing smoking cessation pharmacotherapy, how to use the Refer-to-Quit system and other local cessation referral resources, and a review of NYS health insurance coverage for cessation treatment. The protocol includes the provision of pharmacotherapy consistent with American Dental Association (ADA) recommendations [51]. The initial training presentation will be standardized and conducted by the study co-leaders (DS/JO) and offered onsite at each individual field site. To reinforce the new clinical protocol for documenting and treating tobacco use, each site will receive a 30-minute booster training session approximately three months after the initial training presentation.

#### Chart reminder system

The charting system is designed to remind the clinician to ask those questions required for assessing readiness to quit and to offer each smoker cessation advice. To enhance uptake and sustainability, a tobacco treatment chart prompt will be integrated into the existing chart system (*i.e.*, paper or electronic). Most of the partnering dental health clinics are currently using an electronic health record. Most sites already have billing codes for treating tobacco use, and the remaining sites have agreed to add chart documentation system and billing codes for tobacco dependence.

#### Quitline referral system

Quitlines substantially increase abstinence rates compared to minimal or no counseling [1]. Started in 2000, the NYS Quitline counseled 82,776 current and former smokers in 2012 [52]. The Refer-to-Quit program simplifies the healthcare provider referral process by offering clinical practices a strategy to easily link patients to the NYS Quitline using either a secure online website or a pre-printed fax referral form. The Quitline makes five attempts to reach patients within one week after receiving the fax or online referral. If the Quitline is unable to reach a patient, a letter is sent encouraging the individual to contact the Quitline for assistance. Smoking cessation counselors at the NYS Quitline provide one proactive telephone counseling session and distribute a two-week supply of free nicotine replacement therapy (NRT). A list of local community referrals for high-risk smokers needing more intensive cessation and/or psychosocial services is also provided.

#### Tool kits

All dental providers will receive a Smoking and Oral Health Quit Kit developed specifically for dental care providers by the New York City Department of Health and Mental Hygiene (NYC-DOHMH) [53]. The toolkit contains a call to action letter from the Commissioner of the NYC-DOHMH confirming that treating tobacco use, including prescribing cessation medications, is within dentists’ scope of practice, a provider resource guide for delivery of cessation counseling, patient education booklets describing the oral health effects of tobacco and the benefits of quitting, a pharmacotherapy prescribing information card tailored for dental providers, a summary of oral health in New York City, and a waiting room poster. In addition, our project added Fax/Refer-to-Quit forms to facilitate referral to the NYS Quitline, and a brief version of the PHS Guideline for Treating Tobacco Dependence [1].

#### Tailored clinical pathway

After the baseline data (patient exit interviews and clinic surveys) are collected, we will meet with each site dental director to develop a clinical pathway for treating tobacco use tailored to the site’s current chart system and staffing. The deliverable will be a workflow diagram of the recommended clinical pathway and a detailed table indicating which staff member is responsible for each step in the clinical pathway. For example, we will document who will place the clinical reminder on the charts if there is a paper system, which staff member will offer advice and cessation assistance and specifics about how the Refer-to-Quit system will be implemented in the context of current practice. (*i.e.*, who will help the patient complete the form and who will fax/submit the Quitline referral forms). The new systems and tobacco treatment clinical pathway will be presented during the on-site, staff trainings.

### Arm two: CBP + audit and PF

#### Chart audit and quarterly feedback reports

Clinic sites randomly assigned to arm two will also receive quarterly performance reports on provider delivery of cessation services using chart audit procedures. The research team will work with each of the study sites to develop a system that will allow smokers to be readily identified and placed in a smokers’ registry. This system will vary depending on whether or not the site uses an electronic or paper chart. Quarterly queries of the registry by trained clinic staff will be conducted using a standardized chart audit tool to evaluate documentation of cessation assistance. Each site will be provided and trained on an audit and feedback protocol and the process for disseminating feedback to individual providers. The PF report (see Appendix) will show individual and aggregated clinic performance summaries of targeted 5As provider behaviors benchmarked against other similar clinics and a recommended standard of practice (80%). Quitting assistance will be demonstrated by chart documentation of any of the following provider behaviors: making a referral to the NYS Quitline, or other community-based cessation program; providing cessation counseling; and/or discussing and or prescribing cessation medications [54]. Quarterly reports will be given to the dental director, who will be instructed to circulate and discuss them with the field site’s dental providers within 30 days.

### Arm three: CBP + PF + financial incentive (P4P)

#### Pay for performance

Clinic sites randomly assigned to arm three will receive CBP, quarterly PF reports, and financial incentives for documenting delivery of adherence to clinical practice guidelines. Using the same chart auditing procedures described above, charts of all smokers will be reviewed for documentation of cessation assistance (*i.e.*, prescription given for cessation medication, the provision of brief cessation counseling and/or a referral to the NYS Quitline or other local cessation support programs). Sites will receive $20 for each patient with chart documentation of receiving tobacco cessation assistance. The P4P reimbursement bonus will be offered quarterly with an annual cap of $5,000 per dental clinic. The P4P procedures are guided by published work done by Roski (2003). The incentive amount is also consistent with the current Medicare and New York State Medicaid reimbursement [55,56] schedule for 10 minutes of smoking cessation counseling and represents an amount deemed appropriate by members of our advisory committee representing the ADA and the dental insurance industry.

#### Eligibility and recruitment of field sites

The selection of clinic sites is guided by our intent to insure that findings will be generalizable to real-world dental healthcare settings serving diverse population of smokers. The trial will be conducted in partnership with 18 public health dental clinics employing at least two full-time dentists, not fully adherent to PHS guidelines, able to accept additional compensation if assigned to the P4P arm and serving low-income patients in the New York metropolitan region. For practical (cost and staffing) reasons, clinic sites will be recruited in six successive waves with three sites enrolled per wave. Site randomization will be conducted using the random permuted block method with stratification by clinic volume (small, medium, large). Prior to enrollment, clinics will be categorized as small (100 to 400 patients per week), medium (401 to 750 patients per week) or large (>750 patients per week) based on the number of adult patients seen per week per the dental director. Clinics whose patient volume is less than 100 patients per week will be excluded. Matching the clusters has several practical and statistical advantages. Of particular importance is that matching offers some control for background effects (*e.g.*, cigarette pricing and other public policy changes) contemporaneous of the current trial [57]. Baseline data collection will be completed prior to randomization. If the baseline providers’ assessment reveals that the site assists more than 60% of patients who smoke, then the clinic will be excluded and replaced with an alternate site of similar patient volume.

#### Evaluation plan

The evaluation plan is intended to compare the effectiveness of three promising strategies for implementation of the tobacco use treatment guidelines, to examine multi-level, theory-driven mechanisms (organizational priority, provider beliefs) hypothesized to explain the effectiveness of the three strategies for implementation, and finally to identify multi-level barriers and facilitators that that may influence the implementation of evidence-based tobacco use treatment practices in dental care settings. Mixed methods (*i.e.*, site observational, interview and survey) data will be collected from patients, providers, dental directors, dental charts and Quitline referral data. For clarity, the assessment plan is organized according to following evaluation domains: primary outcomes, secondary outcomes, process outcomes, organizational setting moderators, implementation fidelity, and provider demographics.

### Outcome evaluation (aim one)

#### Primary outcome

To assess the primary outcome of provider adherence to tobacco use treatment guidelines, we will conduct and compare pre- and post-intervention patient exit interviews conducted with eligible smokers at each of the 18 clinic sites. Prior to beginning of the implementation intervention, we will conduct 100 patient exit interviews (n = 1,800 pre-intervention) at each clinic site. Then, after each site has completed the planned intervention, another independent sample of 100 smokers will complete patient exit interviews (n = 1,800 post-intervention). Patient interviews conducted at the point of service are considered to be the optimal non-observational method for measuring provider delivery of outpatient treatment [58,59]. A modified patient exit interview (PEI) [60] will be used as a brief patient-reported measurement tool for the assessment of provider delivery of tobacco use screening and treatment. A PEI index (summary) score is determined by adding the number of tobacco treatment behaviors each patient reports that the provider implemented during the most recent visit. PEI index will scores range from 0 to 8. The questions assess the full spectrum of PHS guideline recommended care (*i.e.*, 5As). For example, patients are asked if their provider advised them to quit, offered cessation counseling and/or discussed cessation medications. The PEI has well-established validity as evidenced by strong correlation with more costly audio-taped assessment of physician-patient interactions (r = 0.67, p < 0.001) and has been commonly used to accurately measure providers’ delivery of tobacco cessation assistance efforts during office practice, and to monitor providers’ adherence to tobacco cessation treatment guidelines in clinical trials. Prior to and approximately nine months following each site’s enrollment, consecutive patients will be approached during their clinic visits by trained research study assistants (RSA), to determine current smoking status and to obtain consent for the PEI. Patient eligibility will include those: age 18 or over; English- or Spanish-speaking; current smokers defined as those who report smoking within the past seven days; New York State residents; and who have an appointment with a dentist or hygienist for routine non-emergent care. Immediately after their dental visit, patients will complete the PEI to assess provider adherence to the PHS Guideline (5As). We expect that most surveys will be administered using a computer tablet with a password protected, web-based data entry portal (REDCap) [61]; however, paper surveys will be available, as needed.

#### Cost analysis

Following recommendations for cost assessment methodology described by Ritzwoller *et al.*, [62] research costs (*e.g.*, labor and other inputs associated with grant administration, IRB approval, manuscript preparation), and direct clinical intervention costs (*e.g.*, dental provider time associated with counseling patients, staff training, IT costs, reimbursement costs) will be estimated. Templates have been developed to capture data prospectively to improve accuracy and precision of cost assessment. The NYS Quitline has estimates of their counseling cost per quit. The costs of the medications taken by the patients as part of their cessation attempt (reported at the three-month telephone survey) will be estimated. In addition, as part of the follow-up assessment, patients will complete the EQ5D, which is a commonly used patient self-report measure of health status. Applicable to a wide range of health conditions, this measure provides a single index value for health status that can be used in the clinical and economic evaluation of healthcare^.^[63].

#### Secondary outcomes

To assess patient utilization of cessation services and smoking abstinence, follow-up telephone interviews, three months post-clinic visit, will be conducted with smokers who complete the post intervention PEIs. A standardized interview will be used to collect patient-reported cessation outcomes including seven-day abstinence and 24-hour quit attempt and use of cessation treatment during the past three months.

### Implementation process evaluation (aim two)

The process evaluation encompasses three areas: TPB processes; Solberg’s construct of Organizational change priority; and acceptability of and satisfaction with the implementation strategies. The multiple data sources (Table 3) include: surveys with participating dentists and dental directors; de-identified data from quarterly chart audits conducted by clinic administrative staff of clinical documentation of tobacco treatment delivery; site observations; de-identified monthly reports from the NYS Quitline summarizing the number of referrals received; and semi-structured interviews with dental clinic directors.

**Table 3:** Evaluation data sources

	**Data source**	**Tool**	**Source**	**Administration**	**Variables**
**Primary outcome**	Patients	Patient Exit Interview (PEI)	[60]	Baseline, nine months following site enrollment	Provider adherence to PHS Guidelines for Treating Tobacco Dependence
**Secondary outcomes**	Patients	Patient Outcomes Survey		Three months after post- PEI	Use of tobacco treatment services, 24-hour quit attempt, Smoking abstinence (7 day point prevalence)
	Research staff and patients	Cost collection template, EQ5D	[62,63]	Ongoing	Costs per quit, QALY, Research and clinical intervention costs
**Process outcomes**	Dental Providers	Provider Attitudes Survey	[42,48,50,64-66]	Baseline, 4.5, and nine months following site enrollment	Provider attitudes, norms, perceived behavioral control, intentions, perceived organizational priority of tobacco use treatment
		Provider Practice Behaviors	[1]	Baseline, 4.5 and nine months following site enrollment	Provider adherence to PHS Guidelines for Treating Tobacco Dependence
		Semi structured Interviews		Nine months following site enrollment	Provider attitudes and practice behaviors
**Organizational setting moderators**	Dental Directors	Organizational Structure Survey	[67-70]	Baseline	Dental staff FTE, patient volume, pay or mix, staff/clinic/patient characteristics
		Change Process Capability Questionnaire	[71]	Baseline	Organizational readiness, change capacity
		Semi structured Interviews		Nine months following enrollment	Organizational implementation and change processes
**Implementation fidelity**	Site observations	Site assessment tool		4.5 and nine months following site enrollment	Pre and post intervention chart and referral systems, workflow
	Dental Chart	Chart audit form		Baseline and Quarterly	Documentation of adherence to tobacco use treatment guidelines
	NYS Quitline	Quitline referral reports		Baseline and Quarterly	Confirmation of changes in Quitline referral patterns
**Provider demographics**	Dental Providers	Provider Demographics		Baseline	Role, degree, years of practice, smoking status, gender

#### Dental director and provider surveys

Measures for four TPB variables (attitudes, subjective norms, perceived behavioral control, and intentions) are based on modifications of selected subscales from established survey tools developed by Park [66], Amemori [64], Francis [50] and Grimshaw *et al.*[48] to assess knowledge and attitudes regarding treatment of tobacco dependence, clinic norms, and perceived behavioral control, and intentions. We will use a modified version of the Perceived Organizational Priority Survey [65] to assess the perceived priority of tobacco use treatment at the dental clinic. We also adapted a tool by Weiner *et al.* to measure additional organizational factors that may influence provider behavior [42]. Semi-structured interviews will be done after completion of the intervention period with the dental director at each of the participating dental clinics. The interviews will focus on site-specific barriers and facilitators of strategy implementation, the process of integrating and customizing the implementation strategy into the clinic site workflow as well as acceptability and satisfaction.

### Baseline organizational characteristics (aim three)

#### Organizational change capacity

The Change Process Capability Questionnaire (CPCQ) will be used to assess baseline measures of organizational capacity to change [71]. The survey measures several domains of change capacity: history of change, plans for continuous organizational refinement, ability to initiate and sustain change, and strategies for process improvement.

#### Organizational structure

At baseline, data on organizational variables shown to influence implementation of provider practice guidelines including number of full-time equivalent staff, insurance payer mix, hospital-based or freestanding clinic, academic affiliation, clinic volume, and staff characteristics will be collected [67-70,72]. Dental directors will complete surveys in order to identify pre-intervention clinical systems and policies (*e.g.*, electronic or paper chart system).

#### Implementation fidelity

We will use several approaches to measure fidelity of the three implementation strategies [73].

#### Current best practices

At baseline, site observations will be conducted to assess clinical processes and workflow (*e.g.*, patient registration, how referrals are handled, who currently screens patients for tobacco use) and these site observations will be repeated at 4.5 and 9 months following site enrollment. During these site visits, use of the dental chart system and Refer-to-Quit forms will be determined. NYS Quitline data will be used as an additional source of information to confirm increases in referral rates in the post intervention period. The NYS Quitline provides monthly reports that include the number of referral forms received by a specific site and provider. In addition, the percentage of clinical staff members who attend the CBP initial and booster training will be recorded.

#### Chart audits and PF

The dental directors will be queried at three, six, and nine months to confirm date of distribution of the quarterly PF reports.

#### Pay for performance

The dental directors assigned to P4P will be also asked at 3, 6, and 9 months to confirm receipt of the quarterly financial incentive earmarked to performance of tobacco treatment interventions.

### Statistical considerations and analytic plan

#### General approach

Eighteen dental clinic sites will be recruited and randomly assigned to one of three implementation strategies in a cluster-randomized design. The general statistical paradigm for assessing outcomes will be based on a Multi-Level Model (MLM) approach [74] (also known as ‘hierarchical linear model’) [8,32,75,76]. MLM adjusts for the clustering effects across multiple levels (patients, providers, dental clinics) of hierarchical data structure. Before the implementation strategies are rolled out, 100 patients per site will be assessed through standardized PEIs to establish the baseline level of dental provider assistance in tobacco cessation. Then each site will be randomly assigned and will receive their intervention condition for nine months. Following the intervention period, 100 new patients from each site will be recruited for assessment of post-intervention changes in provider adherence to the guidelines.

#### MLM approach to address the primary aim

A two-level MLM will test of the effectiveness of the implementation strategies. The primary outcome is the summary score of the PEI assessment of provider assistance (score range 0 – 8). At level one, we will enter each patient’s PEI score of dental provider assistance as a function of intervention and time (pre and post intervention), thereby allowing each site to serve as its own pre-implementation control, an added benefit to minimize an order effect, (*e.g.*, sites that enter the active implementation phase later may derive greater benefits due in part to improved efficiency in logistics coordination) [75-77]. Additional covariates may also be included (*e.g.*, patients’ age, gender, and education).

#### Statistical power and sample size considerations

Statistical power was calculated to ensure sufficient sample size to address two key components of study aims: detect an omnibus effect on the primary outcome of PEI scores across three intervention conditions, against the null hypothesis that providers’ assistance behaviors are equal across intervention conditions; and detect the differences in provider assistance rates (*e.g.*, Quitline referral).

For the primary outcome of provider assistance behaviors, prior data from a recent clinical trial conducted to improve treatment of tobacco dependence in primary care was obtained [78,79]. The investigators compared Intervention (a provider training and practice facilitation program similar to ours) with Usual Care. The Intervention group reported an average PEI provider assistance score of 8.40 (SD = 1.93) versus an average of 6.24 (SD = 2.12) in the Usual Care group, which translates to an estimated effect size of 1.02 (using the larger SD = 2.12). Using this 1.02 effect size, we estimated an 86% statistical power to detect such an effect at a two-sided Type-I error rate of 0.01 (to control for multiple comparisons) for 18 sites (six sites randomized to each intervention condition) and 100 post-intervention patients per site [80].

Statistical power was also estimated for provider assistance behaviors in a generalized estimating equation (GEE) approach. Bentz *et al.*[23] reported that 10.5% of providers randomized to the control condition gave active assistance (*e.g.*, referrals to quitlines); and 20.1% of providers randomized to their feedback condition gave active assistance. Roski *et al.*[33] reported a 31.4% cessation assistance rate for sites randomized to a provider incentive condition. Hence, provider assistance rates are estimated to be 10%, 20%, and 30% (rounded to the nearest decimal point) for the CBP, CBP + PF, and CBP + PF + P4P arms, respectively. Horton’s simulation method [81] was applied to calculate the statistical power of a logistic GEE model. The simulated statistical power was 82% statistical power to detect a 20% versus 10% difference in a GEE model comparing CBP + PF versus CBP alone. The corresponding statistical power for the 30% versus 20% difference between CBP + PF + P4P and CBP + PF is 73%. The statistical power for the larger 30% versus 10% difference between CBP + PF + P4P and CBP alone is 97%. Other parameters in the simulations included a two-sided Type-I error rate of 0.01 to control for multiple comparisons and an intra-class correlation of 0.22, in prior work [23] that closely resembles our trial design.

Using the indicators described in the evaluation plan (See Table 3), relevant process data describing the implementation fidelity of the intervention strategies (CBP, PF, and PFP) will be summarized. All reporting of trial outcomes data will be consistent with cluster randomized CONSORT guidelines [82].

### Ethical review

The Institutional Review Board (IRB) of New York University School of Medicine is the IRB of record and has reviewed and approved the study protocol. Memorial Sloan-Kettering is the Data Coordinating Center and their IRB has also reviewed and approved the study protocol. There is much variation in the policies and procedures for oversight of human subjects’ research among the 18 community-based, dental clinics. In those instances where the dental clinic has an existing affiliation with either an academic center or a private IRB, those IRBs will also provide review and approval. In those instances, where a dental clinic has no affiliation with an IRB, the New York University School of Medicine IRB will provide single project inclusion under their Federal Wide Assurance agreement.

### Trial status

Funded in March 2012, much progress has been made in recruiting community-based dental clinics in New York City, finalizing the evaluation plan, finalizing the provider training plan, finalizing the tool kit for implementation strategies and developing the data management plan for web-based, multi-level data collection. Baseline data collection began in March 2013 and was completed in August 2013 for the first wave of three clinic sites. Fifty-six dental care providers have been enrolled and have completed the baseline provider survey. Four dental directors completed baseline surveys. Staff members at the individual clinic locations have received initial as well as booster training in CBP, and the implementation period is ongoing. Site recruitment and baseline data collection for wave two has begun, and we expect to enroll three additional clinic sites in the Spring of 2014.

## Discussion

Using a cluster randomized controlled trial design and a multi-level conceptual framework combining organizational priority and provider attitudes, norms, perceived behavioral control and intentions, the study protocol will compare the cost and effectiveness of three promising strategies for implementing tobacco treatment guidelines in routine dental care settings serving low-income smokers: Staff training and CBP in implementing PHS Guidelines; CBP + provider PF; and CBP + PF + P4P (provider reimbursement for tobacco cessation treatment delivery). The study builds upon prior research [19,26] and tests practical, multi-level strategies to optimize the implementation of tobacco dependence treatment in dental settings.

The study is innovative in four areas: Dental practices are underrepresented settings in studying implementation of clinical guidelines for treating tobacco dependence; public dental health clinics represent a novel channel for reaching minority and low-income populations who are at greatest risk for tobacco use; the use of a multi-level, theoretical framework for examining provider and practice-level processes for PHS guideline implementation is rare. A recent review of implementation research found that only 10% of prior studies have postulated an explicit theoretical rationale for selection of assessment tools and intervention development [83]. As such, our attention to process data will provide critical information about how the intervention works as well as multi-level barriers and facilitators of implementation, thus providing empirical guidance needed for future adaptations essential for optimizing sustainability and dissemination. Finally our training and clinical assistance model represents a substantive enhancement to the *status quo* of didactic continuing professional education by implementing and evaluating enhanced systems-level strategies to improve the delivery of tobacco treatment in dental clinics.

Some limitations and potential pitfalls need mention. First, although letters of support were obtained from 21 dental directors (18 plus three alternates) and several prominent community public health stakeholders who have pledged their commitment to recruiting additional community-based dental practices, enrollment of dental clinics as field sites may be challenging. Second, it is possible that variation in implementation fidelity will be observed. To reduce this likelihood, training and technical assistance will be standardized and a detailed implementation fidelity measurement plan will assess whether key implementation strategies are delivered. Any variations or adaptations will be documented and these variables will be included in planned analyses. Finally, unforeseen changes in tobacco prevalence (*e.g.*, dual tobacco use) and tobacco policy (*e.g.*, tobacco tax, changes in Medicaid reimbursement, healthcare reform) may affect implementation outcomes. Data on providers’ delivery of cessation assistance will be collected from each site prior to enrollment. This will allow each site to serve as their own control and thereby allow us to analyze for any historical threats to internal validity. Despite limitations, the findings have potential for high impact by identifying best practices for implementing tobacco use treatment in public health dental clinics and providing key stakeholders with the data they need to make decisions regarding implementation of effective tobacco dependence treatment guidelines.

## Abbreviations

CBP: Current best practices; P4P: Pay-for-performance; PF: Performance feedback; PHS: Public health service; IOM: Institute of Medicine; USDHHS: United States Department of Health and Human Services; TBP: Theory of planned behavior; NRT: Nicotine replacement therapy; NYS: New York State; ADA: American Dental Association; NYC-DOHMH: New York City Department of Health and Mental Hygiene; PEI: Patient exit interview; RSAs: Research study assistants; CPCQ: The change process capability questionnaire; MLM: Multi-level model; GEE: Generalized estimating equation; IRB: Institutional review board

## Competing interests

The authors declare that they have no competing interests.

## Authors’ contributions

JO and DS have made equal intellectual contributions to the conceptualization, design and writing of the current protocol. YL assisted in the protocol design and is responsible for the analytic plan. All authors read and approved the final manuscript.

## Authors’ information

Drs. Donna R Shelley and Jamie S Ostroff are Multiple Principal Investigators who have made equivalent scientific contributions to the design of this research protocol and the writing of this manuscript.
